# The Surgical Treatment of Pyorrhea Alveolaris

**Published:** 1917-09

**Authors:** Carl D. Lucas

**Affiliations:** Indianapolis, Ind.


### THE SURGICAL TREATMENT OF PYORRHEA ALVEOLARIS

BY CARL D. LUCAS, D.D.S., INDIANAPOLIS, IND.

[Read before the National Dental Association at its Twentieth Annual Session, Louisville, Ky., July 25-28, 1916.]

Every pyorrhea technician is agreed that the surgical scaling of the precipitate from the cementum of the roots of the affected teeth is the modus operandi of choice for the elimination of pyorrheal foci of infection.

Perhaps each of the many methods of technic heretofore prescribed meet with success at the hands of their sponsors, for the personal equation enters very largely into all surgical procedures.

The first issue we shall insist upon is surgical cleanliness in any method of treatment employed for the removal of calculus.

Before an instrument is used for this purpose the mouth, all interapproximal spaces, all surfaces of crowns and bridges, each infected pocket and the gums should be thoroughly and copiously irrigated with a warm, normal saline solution, thereby mechanically washing the suspended food debris, mouth flora and loosely precipitated mucous plaques from the oral cavity.

High-pressure atomizers, containing so-called antiseptic solutions as sprays for irrigation are contraindicated, on account of the trauma produced upon the affected tissues of low resistance and the danger of a dissemination of the infection.

A gentle, copious irrigation, with at least 16 ounces of a warm physiologic saline solution, directed with a small, sterile glass tube, will not traumatize and the saline solution will stimulate the soft tissues, thereby promoting healing.

A one-quart, sterile metal fountain syringe, suspended two feet above the patient's head, with a glass nozzle the size of the glass point of an eye dropper are the instruments employed for irrigation.

Following the irrigation the preliminary scaling should be done, irrigating the tissues as directed, after each treatment.

From two to six treatments are necessary for the removal of the precipitate en situ, then the crowns and necks of all the teeth in both arches should be thoroughly polished upon all of their surfaces.

Properly shaped brushes should be ordered for the patient and perhaps a demonstration by the operator, as well as explicit instructions as to their uses should be given the patient and a rigid discipline outlined and insisted upon in regard to the personal application of the principles of mouth hygiene.

Since we are endeavoring to eradicate an infectious disease, it is logical that we should employ a definite technic in our treatment to preclude the possibility of the introduction of extraneous infection, therefore all instruments, linens, polishing materials, and the operator's hands should be sterile. A sterile towel should be adjusted to the patient's head to prevent the operator from coming into contact with the hair. The face should be scrubbed with 70 per cent. alcohol before surgical procedure is begun.

During the progress of treatment the instruments should be frequently washed with 70 per cent. alcohol.

A hand bath of a 10 per cent. lysol solution, followed by distilled water should be frequently indulged in by the operator and his assistant, using sterile towels for drying the hands.

Before beginning the surgical planing of the cementum, which is surrounded by an infected pocket, the patient should be placed in a recumbent position and the individual tooth or teeth to be treated should be protected from the saliva by placing sterile cotton rolls upon their labial or buccal and lingual surfaces and a sterile saliva ejector properly adjusted. The teeth should be dried, then bathed with tincture of iodine and dessicated with a jet of warm air.

Their individual surfaces should then be thoroughly and carefully planed, not only free from calculus, but a fraction of a millimeter of the peripherally infected cementum should be cureted away, leaving smooth, finished surfaces.

Normal pericemental fibers are attached to the cementum directly by being definitely engaged in the lamellae of its calcified matrix, therefore, after the pericementum is pathologically detached, the peripheral cementum contains devitalized, necrotic, organic, infected tissue.

We must, therefore, curet the surface cementum deeply enough to eradicate this pathogenic tissue, if we expect to promote healthy granulation in the pocket.

We can not definitely estimate the depth of curetment into the cementum, but our tactile sense must govern our procedure.

Our instruments must be keen edged and care must be exercised not to leave scratches or pits upon the cureted surface. Planing instruments having a pull cut are preferable.

After each surgical treatment the pockets should be irrigated with warm, normal saline solution, flushing the curetment out of the crypts.

Prophylactic treatment must be given at intervals of from one to three months after the case is clinically cured.

Primarily the function of the pyorrhea specialist is the conservation of teeth affected by this disease, but we must not become overly enthusiastic and thereby magnify our ability to eradicate all stages of this infection.

Incipient pyorrhea and some cases of chronic pyorrhea may be successfully arrested and held in check by surgical procedure, followed by systematic prophylactic treatment at frequent intervals, but the teeth should be radiographed in our exaggerated cases to note the depth of pockets containing pyogenic membrane and the amount of osseous destruction.

If the roentgenogram indicates an extensive dissolution of alveolar process, with a detachment of the pericementum to the apical third of the root and the affected tooth is so unstable that it requires a splint for its retention, it is a questionable procedure to attempt any treatment and the conservation of such a tooth. We know that teeth in the most advanced stages of the disease can be retained by splints, but we must first consider the physical welfare of the patients and eliminate all oral focal infection.

Therapeutics will not cure the blind abscess nor destroy its pyogenic membrane, therefore therapeutics will not cure pyorrhea and cause the absorption of its pyogenic membrane.

The blind abscess can be cured only by root resection and curetment of its pyogenic membrane, or the extraction of the tooth and a subsequent curetment of its socket.

In many deep-seated pockets between the approximating surfaces of the teeth in contact, the septum of bone between the teeth is necrotic upon its exposed surface, therefore it becomes necessary to curette the septum to eliminate this irritant. This may be done with burs, mounted stones, or small, sharp bone curets.

We must follow the rules of aseptic surgery as closely as practicable, remove mechanical irritation and establish drainage, if we are to succeed in the promotion of healthy granulation and the eradication of pyorrheal focal infection which in the last analysis is our responsibility to the patients who confide themselves to our care.—*Journal N. D. A.* for July.

